# Case of Presentation of the Anterior Fontanelle

**Published:** 1843-09-02

**Authors:** John A. Elkinton

**Affiliations:** Philadelphia


### CASE OF PRESENTATION OF THE ANTERIOR FONTANELLE.

BY JOHN A. ELKINTON, M. D.

August 4, 1843, at 10 o'clock, P. M., I was called to visit Mrs. F——, in labour with her second child. The membranes ruptured (in my absence from the room) a little before 11 o'clock, the same evening. Labour progressed gradually, with the head presenting, and, as I supposed, with the occiput to the left acetabulum. After waiting some hours, labour advancing slowly, pains continuing all the time feebly, os uteri fully dilated, and pelvis ample, I determined to give ergot, having previously used some tansey or pennyroyal tea which was convenient, without producing any effect. The *powdered* ergot was first administered, and then the *wine* of ergot, neither of which produced any apparent uterine contractions. As the head was moveable, and would occasionally be pressed down by voluntary efforts, in conjunction with pains, so as to appear almost on the point of delivery, I determined to apply the forceps and deliver. The patient becoming exhausted, I commenced the application of the forceps about 4 o'clock in the morning of the fifth. The male blade was readily introduced, but I very soon found it impossible to adjust the female blade.

Without further delay I sent for Professor Hodge, who was soon present.

He attributed the delay and difficulty to a mal presentation of the head, the child presenting the *anterior fontanelle*, instead of the posterior fontanelle to the centre of the pelvis. The position was such that the occiput was somewhat to the left side of the promontory of the sacrum: and the termination of the sagittal suture at the root of the nose, somewhat to the right of the symphisis pubis. Hence two difficulties existed. First, the occipito-frontal, or longitudinal diameter of the cranium corresponded to the sacro-pubic, or short diameter of the superior straight; and secondly, the forehead was anterior, the occiput posterior.

For a favourable delivery, two indications were to be fulfilled. First, to cause flexion of the head, by which the vertex, (or posterior fontanelle) should descend first, and the occipito-bregmatic (or perpendicular) diameter of the head be substituted for occipito-frontal diameter; and second, to cause rotation, so that the occiput might eventually come under the arch of the pubis, and not to the coccyx and perineum. This was accomplished by Dr. Hodge, with the fingers alone; he made pressure behind the symphisis, on the right side of the os frontis, pushing it upwards so as to cause flexion of the head, and at the same time to the right and backwards, so as to direct the face towards the hollow of the sacrum, and of course the occiput from the sacrum to the left acetabulum and symphisis pubis.

Having thus effected this important change in the position of the child, I was requested to take charge of the patient, and in a few minutes she was delivered of a large and healthy child, without further interference or artificial aid.

The object in this brief notice is to record the peculiarity of the presentation, and to confess the difficulty I have occasionally experienced in *diagnosis.* Who can *positively* determine the exact presentation of the child's head, in the early stage of labour? This case proves the importance of accurate distinctions in all varieties of presentation.

*Philadelphia*, Aug. 21, 1843.

